# A complex teamwork intervention in a surgical ward in Norway

**DOI:** 10.1186/s13104-019-4619-z

**Published:** 2019-09-14

**Authors:** Oddveig Reiersdal Aaberg, Marie Louise Hall-Lord, Sissel Iren Eikeland Husebø, Randi Ballangrud

**Affiliations:** 1 Department of Health Science, Faculty of Medicine and Health Sciences, Norwegian University of Science and Technology, Teknologivegen 22, 2815 Gjøvik, Norway; 2 Department of Quality and Health Technology, Faculty of Health Sciences, University of Stavanger, Kjell Arholmsgate 41, 4036 Stavanger, Norway; 3 Department of Health Sciences, Faculty of Health, Science and Technology, Karlstad University, Universitetsgatan 2, 651 88 Karlstad, Sweden; 4 Department of Surgery, Stavanger University Hospital, Gerd-Ragna Bloch Thorsens Gate 8, 4011 Stavanger, Norway

**Keywords:** Implementation, Interprofessional, Intervention, Patient safety, Surgical ward, Team training, TeamSTEPPS

## Abstract

**Objectives:**

Interprofessional team training has a positive impact on team behavior and patient safety culture. The overall objective of the study was to explore the impact of an interprofessional teamwork intervention in a surgical ward on structure, process and outcome. In this paper, the implementation of the teamwork intervention is reported to expand the understanding of the future evaluation results of this study.

**Results:**

The evidence-based Team Strategies and Tools to Enhance Performance and Patient Safety (TeamSTEPPS) program was implemented in three phases according to the program’s implementation plan, which are built on Kotter’s organizational change model. In the first phase, a project group with the leaders and researchers was established and information about the project was given to all health care personnel in the ward. The second phase comprised 6 h interprofessional team training for all frontline health care personnel followed by 12 months implementation of TeamSTEPPS tools and strategies. In the third phase, the implementation of the tools and strategies continued, and refresher training was conducted.

*Trial registration* Trial registration number (TRN) is ISRCTN13997367. The study was registered retrospectively with registration date May 30, 2017

## Introduction

In today’s specialized, complex and pressurized healthcare, harm caused by adverse events during hospital care are acknowledged as a serious threat to patient safety, with human factors as a central issue [1, 2]. Enhancing teamwork and communication between interprofessional healthcare personnel has a positive impact on patient safety in healthcare systems founded on human factors principles [1]. Interprofessional teamwork comprises different healthcare professions, which share a team identity and work closely together in an integrated and interdependent manner to solve problems and deliver healthcare services [3].

A considerable number of adverse events are related to surgical treatment [4, 5]. A microsystem, such as a surgical ward, has the greatest opportunity to improve work processes as part of interprofessional teamwork [6, 7]. There is limited amount of research on interprofessional team training in the context of surgical wards [8, 9], and in this paper the implementation of the teamwork intervention in a surgical ward will be described. The study protocol has been previously published [10].

## Main text

Team training is an effective method to improve frontline healthcare personnel’s teamwork competencies [9]. Team training is defined as “a set of tools and methods that form an instructional strategy”, and is a methodology designed to educate team members with the competencies necessary for optimizing teamwork [11]. Teamwork competencies refer to the attitudes, behaviors and cognitions necessary for effective teamwork [12]. In this context, the attitudes are the affective attributes essential for effective team performance, behaviors are the skills and procedures needed for teamwork and cognitions are the necessary elements of knowledge and experience necessary for effective teamwork [12]. Interprofessional team training in hospitals has a positive impact on team behavior [9, 13], patient safety culture [14] and patient outcome [9].

Team Strategies and Tools to Enhance Performance and Patient Safety (TeamSTEPPS) [15] was chosen as the team training program in this study. This generic program is one of a few standardized training and research-based programs that address the impact of human factors on healthcare teams [16]. The program aims to optimize patient safety and the quality of care by enhancing team structure and four teamwork competencies; communication, leadership, situation monitoring, mutual support [17–19]. Each of the four teamwork competencies has a set of tools or strategies that the interprofessional team members are supposed to utilize to ensure effective teamwork [17, 20]. The AHRQ gave permission to translate and use the program in Norway.

Despite previous research which shows that interprofessional team training interventions improve the quality of clinical practice, there is little knowledge about its impact on hospital wards. The overall objective of the study was therefore to explore the impact of an interprofessional teamwork intervention in a surgical ward on structure, process and outcome. Here, we report on the implementation of the TeamSTEPPS program as additional data to facilitate a better understanding of the evaluation results of this study.

### Research setting and sample

The interprofessional teamwork intervention was carried out in a surgical ward at hospital in eastern Norway. The ward was selected for convenience, and based on the management`s interest to participate in the study. The target group was frontline healthcare personnel consisting of physicians, registered nurses and nursing assistants working at the ward. A consecutive sample of elective and emergency patients with the inclusion criteria being 18 years or older, understanding Norwegian, and being in a mental and physical health condition that made it ethically justifiable to participate, were included in the study. Furthermore, healthcare personnel in a surgical ward at a hospital in southern Norway constituted the control group.

### Description of the intervention

The intervention was initiated by a research group consisting of four researchers from two universities in Norway. Two of the researchers and four of the leaders at the ward had attended a TeamSTEPPS master training. The TeamSTEPPS program and teaching materials were translated into Norwegian by a translation agency, and approved by three faculty members. The intervention was conducted according to the recommended TeamSTEPPS implementation plan [17], which is built on John Kotter’s change model with eight steps for organizational change [21]. The TeamSTEPPS intervention is described in three phases, with Kotter’s eight steps incorporated in the different phases. The study period lasted for 12 months after the initial interprofessional team training.

#### Phase I. Setting the stage and deciding what to do—assessment and planning

Multiple actions occurred to assess organizational readiness for a TeamSTEPPS initiative [17, 21]. After collection of the assessment profile of the surgical ward, the leaders decided that the ward was ready for the TeamSTEPPS intervention. A comprehensive planning for an interprofessional TeamSTEPPS training and implementation was jointly conducted by the leaders and the researchers. In advance of the team training, the physicians and nursing staff attended information meetings organized by the researchers.

#### Phase II: Make it happen—training and implementation

The onset for the TeamSTEPPS intervention was 6 h of compulsory interprofessional team training conducted for all frontline healthcare personnel during work hours (n = 41). TeamSTEPPS leaflets and pocket guides were distributed to all healthcare personnel. The team training was conducted by the leaders of the ward in collaboration with two of the researchers (RB and ORA), and completed over 3 days (three interprofessional groups) during 3 weeks in May 2016. The team training consisted of didactics, videos, role-play and high-fidelity simulation training with debriefing sessions. The first lecture aimed to create a sense of urgency (Kotter’s step 1) by presenting the Sue Sheridan video [17] and by presenting the hospital`s reports of adverse events. The two simulation sessions consisted of two scenarios: “A postoperative urology patient with infection” and “A postoperative gastroenterological patient with acute deterioration”, both with a focus on communication and teamwork. At the end of the course, all healthcare personnel were asked to identify patient safety issues in the ward, and to suggest TeamSTEPPS tools to solve the problem. Moreover, immediately after the training the participants responded to “The TeamSTEPPS Course Evaluation Survey” [22] to evaluate the training and learning outcomes. The 6 h of team training was accredited for continuing education by for general surgery by the Norwegian Medical Association and for clinical advancement by the Norwegian Nurse Organization.

The initial team training was followed by an implementation phase in the intervention ward. A Change Team was established (Kotter’s step 2) consisting of multi-professional healthcare personnel from the ward (two registered nurses, two nursing assistants and four physicians), in addition to the Chair of the surgical department, a former patient and a researcher serving as a coach (ORA). The Change Team served as a guiding coalition and was led by the Nurse Unit Manager. A vision of “Zero errors” (0 patient harm errors) was set, and an action plan was developed based on the identified patient safety risk areas in the ward and approved by the Chair of the surgical department (Kotter’s step 3). The action plan was communicated in unit staff meetings and by email to all employees (Kotter’s step 4). The leaders empowered a broad-based action to make the implementation as smooth as possible, and to remove obstacles that could undermine the changes (Kotter’s step 5). Posters with explanations of the TeamSTEPPS tools were placed in working stations at the ward. In addition, posters for patients and visitors were placed in the ward corridors, with a request to speak up if they perceived something that might be a threat to patient safety. One of the authors (ORA) coached the implementation by giving and gathering input from site visits and e-mail communications with the leaders and the Clinical Nurse Specialist, and as a member of the Change Team throughout the study period.

The Nurse Unit Manager and the Clinical Nurse Specialist led the implementation of tools and strategies. Five TeamSTEPPS tools were implemented during the first 6 months of the study period (see Table 1). Each tool was launched as “The tool of the month”, and communicated in the weekly newsletters sent to the nursing staff and physicians. The introduction of new tools was marked in inventive ways. An example of structural changes that followed the training were implementation of interprofessional huddles held beside the patient safety whiteboard after the daily interprofessional rounds.

**Table 1:** Overview of team training and implemented TeamSTEPPS tools and strategies—and their related key principles

Year Month	Tools and strategies Team competencies	Explanations of the team training and the tools and strategies implemented	Profession group Implementation
2016 May	Patient safety and TeamSTEPPS five key principles	Six hours of compulsory team training: didactics, discussions, role-play and high-fidelity simulation training	All healthcare personnel
2016 May	Closed-loop Communication	To ensure that information conveyed by the sender is understood by the receiver as intended. The sender has to ensure verification from the receiver	All healthcare personnel Critical information—especially regarding orally medicating orders
2016 June	SBAR Communication	A technique for communicating critical information that requires immediate attention and action concerning a patient’s condition. SBAR: S = Situation B = Background A = Assessment R = Request/Recommendation	All healthcare personnel For example, by a deteriorating patient
2016 August	Briefs Leadership	Sharing the plan: Short session prior to start? To share the plan, discuss team formation, assign roles and responsibilities, establish expectations and climate, anticipate outcomes and likely contingencies	Nursing staff Start of every shift
2016 September	Huddles Leadership	Monitoring and modifying the plan: Ad hoc meeting to reestablish situational awareness, reinforce plans already in place and assess the need to adjust the plan	All healthcare personnel Daily after rounding by the patient whiteboard—the rounding physician and nursing staff—lead by a registered nurse
2016 October	Cross monitoring Situation monitoring	A harm error reduction strategy that involves: (1) Monitoring actions of other team members; (2) Providing a safety net within the team; (3) Ensuring that mistakes or oversights are caught quickly and easily, and (4) “Watching each other’s back”	Registered nurses Mandatory control by two registered nurses with intravenous I.V. medication administration
2016 November	TeamSTEPPS 4 team competencies	TeamSTEPPS refresher training; classroom team training	Nursing staff (75 min) and physicians (20 min)
2017 January	Debriefs Leadership	Reviewing the Team’s Performance. Informal information exchange session designed to improve team performance and effectiveness through lessons learned and reinforcement of positive behaviors	Nursing staff Once a week with Unit Nurse Manager
2017 January	Task assistance Mutual support	Helping others with tasks builds a strong team. Key strategies include: (1) Team members protect each other from work overload, (2) Effective teams place all offers and requests for assistance in the context of patient safety, and (3) Team members foster a climate where it is expected that assistance will be actively sought and offered	Nursing staff Distribution of workload, number of patients
2017 February	STEP Situation Monitoring	A tool for monitoring situations in the delivery of healthcare. Status of the patient (S), Team members (T), Environment (E), Progress toward the goal (P)	Nursing staff Updated electronic care plan
2017 March	Two challenge rule Mutual support	Empowers all team members to “stop the line” if they sense or discover an essential safety breach. When an initial assertive statement is ignored: (1) It is the team members’ responsibility to assertively voice concern at least two times to ensure that it has been heard, (2) The team member being challenged must acknowledge that concern has been heard, and (3) If the safety issue still hasn’t been addressed, the team member has to take a stronger course of action and utilize supervisor or chain of command	All healthcare personnel Anyone: Speak-up until heard when seeing a situation that may threaten patient safety
2017 May	TeamSTEPPS 4 team competencies	TeamSTEPPS refresher training; classroom team training	Nursing staff (75 min)
2017 May	I-PASS Communication	The transfer of information (along with authority and responsibility) during transitions in care across the continuum. It includes an opportunity to ask questions, clarify, and confirm. I = Illness severity, P = Patient summary, A = Action list, S = Situation awareness and contingency planning, S = Synthesis by receiver	Nursing staff Handoffs with focus on patient safety risks

The frontline healthcare personnel and their leaders celebrated short-term wins together (Kotter’s step 6). Five months after the initial team training, the master-trained Unit Nurse Manager and the Clinical Nurse Specialist organized 75 min TeamSTEPPS refresher training for the nursing staff. The master-trained Chief Surgeon conducted a 20 min refresher training for the physicians.

#### Phase III: Make it stick—sustainment

The implementation of the tools and structural changes continued into the sustainment phase, with five more tools implemented during the next 6 months. After 11 months, one more 75 min TeamSTEPPS refresher training was conducted for all the nursing staff. The leaders and the frontline healthcare staff used the tools in their daily work, and the changes were consolidated (Kotter’s step 7). They anchored the change to let the change effort to become a lasting part of the organizational culture (Kotter’s step 8). This step is the final leg of Kotter’s leading change model. The implementation period was 12 months.

**Fig. 1:**
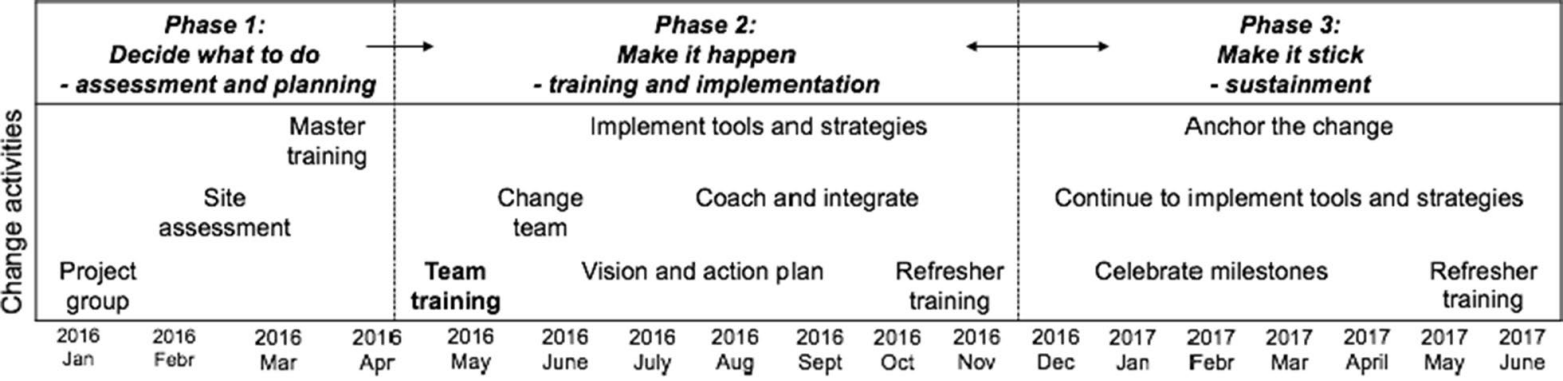
Overview of the intervention

An overview of the intervention is illustrated in Fig. 1, and more details are given in Table 1.

### Evaluations

The intervention will be evaluated by qualitative focus group interviews with healthcare personnel, and by quantitative questionnaires administered to healthcare personnel and patients. To study changes in patient safety culture, the Hospital Survey of Patient Safety Culture Questionnaire (HSOPS) [23, 24] will be used. To investigate the effect on the intervention on teamwork, the TeamSTEPPS Teamwork Perceptions Questionnaire (T-TPQ) [25, 26], the Collaboration and Satisfaction About Care Decisions in Teams Questionnaire (CSACD-T) [27, 28], and the TeamSTEPPS Teamwork Attitude Questionnaire (T-TAQ) [29, 30] will be used. To explore patients experiences with the quality of care, the Quality from Patient’s Perspective (QPP) questionnaire will be used [31]. Moreover, anonymous patient data from hospital complication register, Global Trigger Tool data will be used to evaluate the effect of the intervention. Table 2 gives an overview of the evaluation methods in relation to the specific objectives, design and sample. For further details see the published study protocol [10].

**Table 2:** Overview of evaluation methods in relation to the specific objectives, design and sample

Specific objectives	Design	Sample	Evaluation methods
To explore the impact of an inter-professional teamwork intervention in a surgical ward with regard to teamwork and patient safety culture	Quasi-experimental—controlled before-and after	Healthcare personnel from the intervention ward and a control ward	T-TPQ, T-TAQ, CSACD-T and HSOPS questionnaires before the intervention and after 12 months of intervention
To describe inter-professional team members’ perception of teamwork and the impact of implementation of a teamwork intervention in a surgical ward	Qualitative descriptive	Healthcare personnel from the intervention ward	Focus group interviews before the intervention and after 6 months and 12 months of intervention
To explore patients’ perception of quality of care in relation to an inter-professional teamwork intervention in a surgical ward	Quasi-experimental—uncontrolled before and after	Patients from the intervention ward	QPP questionnaire before the intervention and after a period of 6 months and 12 months of intervention
To explore the impact of an inter-professional teamwork intervention in a surgical ward regarding patient outcome and adverse events	Quasi-experimental—time-series	Hospital complication registries and data from patients’ medical records from the intervention ward	Complication data, LOS, Global Trigger Tool data—before and during the 12-months intervention period

*T-TPQ* TeamSTEPPS Teamwork Perceptions Questionnaire, *T-TAQ* TeamSTEPPS Teamwork Attitude Questionnaire, *CSACD-T* Collaboration and Satisfaction About Care Decisions in Team Questionnaire, *HSOPS* Hospital Survey on Patient Safety Culture Questionnaire, *QPP* Quality from Patient’s Perspective Questionnaire

## Limitations

The main limitations of these studies are as follows: (1) the change of the Unit Nurse Manager during the study period might have influenced the results, (2) the research team had limited control of the intervention, and (3) the convenience sample of the intervention ward could be a possible bias.

## Data Availability

Data sharing is not applicable to this paper, as no datasets or analyses were generated during the current paper.

## Abbreviations

AHRQ: Agency for Healthcare Research and Quality
TeamSTEPPS: Team Strategies and Tools to Enhance Performance and Patient Safety

## References

[1] Clinical Human Factors Group. Implementing Human Factors in Healthcare—‘Taking further steps’. ‘How to’ Guide to Human Factors 2013. https://improvementacademy.org/documents/Projects/human_factors/Implementing-human-factors-in-healthcare-How-to-guide-volume-2-FINAL-2013_05_16.pdf. Accessed 7 May 2019.

[2] WHO. Human factors in patient safety. Review of topics and tools. In: Report for methods and measures working, ed. Geneva: WHO; 2009.

[3] Reeves S (2010). Interprofessional teamwork for health and social care.

[4] de Vries EN (2008). The incidence and nature of in-hospital adverse events: a systematic review. Qual Saf Health Care.

[5] Zegers M (2011). The incidence, root-causes, and outcomes of adverse events in surgical units: implication for potential prevention strategies. Patient Saf Surg.

[6] Mohr JJ, Batalden PB (2002). Improving safety on the front lines: the role of clinical microsystems. BMJ Qual Saf.

[7] McGinnis JM (2013). Best care at lower cost: the path to continuously learning health care in America.

[8] Aaberg OR, Wiig S. Interprofessional team training in hospital wards: a literature review. In: European safety and reliability conference (ESREL). Portoroz: CRC Press; 2017.

[9] Hughes AM (2016). Saving lives: a meta-analysis of team training in healthcare. J Appl Psychol.

[10] Ballangrud R (2017). STUDY PROTOCOL Open Access“Teamwork in hospitals”: a quasi-experimental study protocol applying ahuman factors approach. BMC Nurs.

[11] Salas E, Cooke NJ, Rosen MA (2008). On teams, teamwork, and team performance: discoveries and developments. Hum Fact.

[12] Salas E, Salas E, Goodwin G, Burke C (2008). The wisdom of collectives in organizations: an update of the teamwork competencies. Team effectiveness in complex organizations: cross-disciplinary perspectives and approaches.

[13] O’dea A, O’connor P, Keogh I (2014). A meta-analysis of the effectiveness of crew resource management training in acute care domains. Postgrad Med J.

[14] Sacks GD (2015). Teamwork, communication and safety climate: a systematic review of interventions to improve surgical culture. BMJ Qual Saf.

[15] King HB, Henriksen K (2008). TeamSTEPPS™: Team Strategies and Tools to Enhance Performance and Patient Safety. Advances in patient safety: new directions and alternative approaches.

[16] Gross B (2019). Crew resource management training in healthcare: a systematic review of intervention design, training conditions and evaluation. BMJ Open.

[17] TeamSTEPPS 2.0 2014. https://www.ahrq.gov/teamstepps/instructor/index.html. Accessed 12 Aug 2019.

[18] Alonso A (2006). Reducing medical error in the military health system: how can team training help?. Hum Resourc Manag Rev.

[19] Alonso A, Dunleavy D, Salas E, Frush K (2012). Building teamwork skills in health care: the case for coordination and communication competences. Improving patient safety through teamwork and team training.

[20] King HB, et al. TeamSTEPPS: Team Strategies and Tools to Enhance Performance and Patient Safety. In: Henriksen K, et al. editors. Advances in patient safety: new directions and alternative approaches (vol. 3: performance and tools). Rockville: Agency for Healthcare Research and Quality (US); 2008. 21249925

[21] Kotter JP (2012). Leading change.

[22] TeamSTEPPS 2.0. Course Evaluation. 2014 Content last reviewed March 2014. https://www.ahrq.gov/teamstepps/instructor/reference/smpcefm.html. Accessed 24 Feb 2019.

[23] Sorra J, Dyer N (2010). Multilevel psychometric properties of the AHRQ hospital survey on patient safety culture. BMC Health Serv Res.

[24] Olsen E, Øvretveit J, Sousa P (2008). Reliability and validity of the hospital survey on patient safety culture at a Norwegian hospital. Quality and safety improvement research: methods and research practice from the International Quality Improvement Research Network.

[25] American Institutes for Research. TeamSTEPPS^®^ Teamwork Perceptions Questionnaire Manual. 2010.

[26] Ballangrud R, Husebø SE, Hall-Lord ML (2017). Cross-cultural validation and psychometric testing of the Norwegian version of the TeamSTEPPS^®^ teamwork perceptions questionnaire. BMC Health Serv Res.

[27] Baggs JG (1994). Development of an instrument to measure collaboration and satisfaction about care decisions. J Adv Nurs.

[28] Aaberg OR (2019). Collaboration and Satisfaction About Care Decisions in Team questionnaire—psychometric testing of the Norwegian version, and hospital healthcare personnel perceptions across hospital units. Nursing Open.

[29] Ballangrud R, Husebø SE, Hall-Lord ML (2019). Cross-cultural validation and psychometric testing of the Norwegian version of TeamSTEPPS Teamwork Attitude Questionnaire. J Interprof Care.

[30] Baker DP (2010). Assessing teamwork attitudes in healthcare: development of the TeamSTEPPS teamwork attitudes questionnaire. Qual Saf Health Care.

[31] Larsson B, Larsson G (2002). Quality from Patient’s Perspective questionnaire: development of a short form of the Quality from the Patient’s Perspective (QPP) questionnaire. J Clin Nurs.

[32] The World Medical Association. Declaration of Helsinki—Ethical Principles for Medical Research Involving Human Subjects 2013. Accessed 15 May 2019.

